# Opposite Role of Tumor Necrosis Factor Receptors in Dextran Sulfate Sodium-Induced Colitis in Mice

**DOI:** 10.1371/journal.pone.0052924

**Published:** 2012-12-28

**Authors:** Ke Wang, Gencheng Han, Yan Dou, Yi Wang, Guijun Liu, Renxi Wang, He Xiao, Xinying Li, Chunmei Hou, Beifen Shen, Renfeng Guo, Yan Li, Yanchun Shi, Guojiang Chen

**Affiliations:** 1 Department of Immunology, School of Basic Medical Sciences, Central South University, Changsha, P. R. China; 2 Department of Immunology, Institute of Basic Medical Sciences, Beijing, P. R. China; 3 Department of Gastroenterology, General Hospital of PLA, Beijing, P. R. China; 4 Center of Molecular Biology, Inner Mongolia Medical College, Hohhot, P. R. China; 5 InflaRx GmbH, Jena, Germany; Juntendo University School of Medicine, Japan

## Abstract

Tumor necrosis factor-α (TNF-α) is a key factor for the pathogenesis of inflammatory bowel diseases (IBD), whose function is known to be mediated by TNF receptor 1 (TNFR1) or 2. However, the precise role of the two receptors in IBD remains poorly understood. Herein, acute colitis was induced by dextran sulfate sodium (DSS) instillation in TNFR1 or 2−/− mice. TNFR1 ablation led to exacerbation of signs of colitis, including more weight loss, increased mortality, colon shortening and oedema, severe intestinal damage, and higher levels of myeloperoxidase compared to wild-type counterparts. While, TNFR2 deficiency had opposite effects. This discrepancy was reflected by alteration of proinflammatory cytokine and chemokine production in the colons. Importantly, TNFR1 ablation rendered enhanced apoptosis of colonic epithelial cells and TNFR2 deficiency conferred pro-apoptotic effects of lamina propria (LP)-immune cells, as shown by the decreased ratio of Bcl-2/Bax and enhanced nuclear factor (NF)-κB activity.

## Introduction

Tumor necrosis factor (TNF) plays a crucial role in immune response at physiological and pathological states, which is based on its pleiotropic function on differentiation, growth and apoptosis of both immune and non-immune cell populations [1], [2]. Numerous studies have demonstrated that TNF is tightly implicated in the pathogenesis of inflammatory diseases and autoimmunity such as rheumatoid arthritis, multiple sclerosis and inflammatory bowel diseases (IBD) [3]. It is well established that the biological function of TNF is exerted through binding as a trimer to either TNF receptor (TNF-R) 1 or 2. TNF-R1, which is expressed on most cell types, contains a death domain that mediates apoptotic signaling through caspase activation and activates nuclear factor (NF)-κB, resulting in transcription of proinflammatory cytokines and chemokines as well as anti-apoptotic peptides [4]. TNF-R2, which is expressed predominantly by haematopoietic cells, lacks the death domains but delivers apoptotic signaling through the kinase receptor-interacting protein (RIP) [5].

Increasing data have been shown to support the intimate relationship of TNF-R expression with the pathogenesis of IBD. During active stages of the disease, TNF-R2 expression by colonic epithelial cells is increased in patients suffering from IBD and in mice with experimental colitis [6], [7]. Furthermore, the polymorphisms in the TNF-R2 gene have been reported to be associated with a higher susceptibility to Crohn’s disease (CD) [8]. These observations strongly support a disease-promoting role of TNF signaling via TNF-R2 during IBD development. However, a study demonstrates recently that the transfer of colitogenic CD4^+^CD45^hi^TNF-R2^−/−^ T cells into RAG−/− recipients leads to acceleration of the onset of overt disease and to aggravated severity of intestinal inflammation [9], indicating an opposite effect of TNF-R2 expression by CD4^+^ T cells on the course of colitis. The paradoxicality on the role of TNF signaling via TNF-R1 in IBD also exists. Ebath et al. [10] reported that TNF-R1-deficient C57Bl/6 mice had more weight loss and increased mortality after trinitrobenzene sulphonic acid (TNBS) instillation. In contrast, Nakai et al. [11] demonstrated that TNF-R1 ablation attenuated tissue damage after TNBS, which was related to reduced NF-κB activity. Our recent study has shown that TNF signaling via TNF-R1or TNF-R2 play a pathogenic role in TNBS-induced colitis [12]. Overall, these studies underline the complexity of TNF bioactivity via TNF-R1 or 2 during the onset and perpetuation of intestinal inflammation, which may be affected by different TNF-R expression patterns and distinct colitis models used.

The studies on the function of TNF signaling via TNF-R1 or 2 on the course of DSS-induced colitis, which is another widely used murine model of IBD and closely resembles UC, is relatively few. Recently, Stillie et al. reported that, although ablation of TNF-R1or 2 had an effect on some parameters of DSS colitis in C57Bl/6 mice, TNF signaling via either of its receptors appeared to play a redundant role in the pathology of intestinal inflammation [13]. The exact role of TNF signaling via TNF-R1 or 2 in the course of DSS colitis in other strains and underlying mechanisms, however, remains poorly understood. In this study, we investigated the effect of TNF-R1 or 2 knockout on DSS-induced acute colitis in BALB/c strains. Ablation of TNF-R1 or 2 had opposite influence on the course of colitis. That is, TNF-R1 deficiency accelerated the onset of overt diseases, while TNF-R2 knockout attenuated the severity of colitis. This disagreement can be resolved by that TNF-R1 deficiency led to augmented production of proinflammatory cytokines in the lesions, while TNF-R2 knockout did not. Furthermore, enhanced apoptosis of colonic epithelial cells (CEC) was found in TNF-R1-deficient mice after DSS. In contrast, immune cells in the lamina propria (LP) had a trend to programmed death in TNF-R2-knockout rodents after DSS.

## Materials and Methods

### Animals

BALB/c wild type (WT) mice were purchased from Jackson Laboratory (Bar Harbor, Maine). TNFR1^−/−^ and TNFR2^−/−^ mice from Dr. Zhihai Qin [National Laboratory of Biomacromolecules, Institute of Biophysics, Chinese Academy of Sciences, Beijing, China] were backcrossed for 12 generations onto the BALB/c background [14]. Animals were housed in specific pathogen-free conditions with an alternating light/dark cycle. All experiments were performed using 6- to 8-week-old male mice. Care, use and treatment of mice in this study was in strict agreement with international guidelines for the care and use of laboratory animals and approved by Animal Ethics Committee of Institute of Basic Medical Sciences.

### Colitis Induction

Acute colitis was induced in mice by adding 3% DSS (MW 36, 000–50, 000; MP Biochemicals, USA) to their drinking water and allowing them drink ad libitum, starting from day 0 for 8 days. Weights were measured before induction of colitis and daily thereafter. At the end of the experiments mice were sacrificed by cervical dislocation under anesthesia.

### Histology

Colons removed from mice at indicated time of death were fixed in 10% neutral-buffered formalin solution and then embedded in paraffin, cun into tissue sections, and stained with hematoxylin and eosin. Stained sections were examined for evidence of colitis using as criteria: the presence of lymphocyte infiltration, elongation and/or distortion of crypts, frank ulceration, and thickening of the bowel wall. The degree of inflammation on microscopic cross-sections of the colon was graded semiquatitatively from 0 to 4 (0, no evidence of inflammation; 1, low level of lymphocyte infiltration with infiltration seen in <10% high-power field (hpf), no structural changes observed; 2, moderate lymphocyte infiltration with infiltration seen in 10–25% hpf, crypt elongation, bowel wall thickeningwhich does not extend beyond mucosal layer, no evidence of ulceration; 3, high level of lymphocyte infiltration with infiltration seen in 25–50% hpf, high vascular density, thickening of bowel wall which extends beyond mucosal layer; and 4, marked degree of lymphocyte infiltration with infiltration seen in >50% hpf, high vascular density, crypt elongation with distortion, transmural bowel wall thickening with ulceration).

### Isolation of Colonic Epithelial Cells and Lamina Propria-immune Cells

The entire length of colon was opened longitudinally, washed with PBS, washed with PBS containing 100 µg/ml gentamycin, and cut into small pieces. The dissected mucosa was incubated with Ca^2+^, Mg^2+^-free HBSS supplemented with 10% fetal bovine serum, 5 mM EDTA, 15 mM HEPES, 100 µg/ml gentamycin under shaking at 37°C for 20 min then repeated for 4 times. The supernatants containing colonic epithelial cells were centrifuged at 800 g for 10 min at 4°C. the deposit was collected as colonic epithelial cells (purity>80%, identified by CK-18 staining). The remaining was washed with HBSS, followed by digestion in RPMI 1640 medium containing 100 U/ml collagenase D (Roche Diagnostics) under shaking at 37°C for 1 h then repeated for 2 times. The total of digested supernatants was centrifuged at 800 g for 10 min at 4°C and washed with PBS. The pellet was layered on a 40%/100% Percoll gradient (Pharmacia) and spun at 1,800 rpm for 5 min to collect the lymphocyte-enriched population at the 40%/100% Percoll interface as LP-immune cells.

### Detection of Apoptotic Cells

For detection of apoptotic cells in tissues, labeling of degraded DNA specific to apoptotic cells was performed using a modification of the terminal deoxynucleotidyl transferase-mediated deoxyuridine triphosphate nick end-labeling (TUNEL) technique by application of the *in situ* cell death detection kits (Roche Diagnostics, Indianapolis, IN) according to the manufacturer’s instructions. In this method, residues of digoxigenin-labeled nucleotides are added to the ends of DNA fragments by terminal deoxynucleotidyl transferase, then peroxidase-labeled antidigoxigenin antibodies and 3,3′-diaminobenzidine are added in turn; the latter are oxidized by the bound peroxidase to generate visible signals from the apoptotic cells.

### Colon Homogenates

A 1 cm segment was divided from the distal 4 cm of the harvest colon. Wet weight was recorded separately for the whole distal 4 cm and the portion taken for homogenation. Colon tissue samples were homogenized in PBS containing a cocktail of protease inhibitors (1 µl to 20 mg of tissue according to the manufacturer’s protocol, Roche) with a Polytron homogenizer and centrifuged at 12 000 *g* for 10 min. The supernatants were stored at −20°C until used for ELISA analysis.

### Determination for Colon Edema

Colons were dissected at indicated timepoint after TNBS. A piece of the affected colon was then collected, weighed, placed in an 80°C oven for 24 h, then reweighed, and the wet-to-dry weight ratio was determined as a measure of colon edema [15].

### Measurement of Myeloperoxidase Activity

Tissue MPO activity was determined by a standard enzymatic procedure as described previously [16], with minor modifications. Briefly, after the samples has been weighed, a tissue sample (approximately 300 mg) was homogenized in a buffer (0.5% hexadecyltrimethylammonium bromide in 50 Mm potassium phosphate buffer, pH 6.0) three times for 30 s each on ice. The sample was centrifuged at 20,000 g for 20 min at 4°C and the supernatant was collected. The supernatants (100 µl) was then added to 2.9 ml of 50 mM phosphate buffer (pH 6.0) containing 0.167 mg/ml O-dianisidine hydrochloride and 0.0005% hydrogen peroxide, and absorbances were measured using a spectrometer at 25°C. Results for colon MPO content were converted to absorbance units per gram of tissue.

### Immunoblotting

Samples were homogenized and sonicated in RIPA lysis buffer (Santa Cruz Biotechnology Inc.), supplemented with protease inhibitors. After centrifugation at 20,000 *g* for 15 min, 30 µg of the supernatants were separated on 10% SDS-polyacrylamide gel and transferred onto an Immunobilon-P Transfer membrane (Millipore). After being blocked with 5% skim milk, the membrane was incubated with antibodies to Bcl-2 (1∶1000), Bax (1∶1000), or IκBα (1∶1000). Rabbit anti-actin antibody (1∶1000) was used as an internal control. ImmunoPure peroxidase-conjugated anti-rabbit IgG were used as secondary antibodies. The blotted membrane was then treated with the Super Signal West Dura Extended Duration Substrate (Pierce Biotechnology Inc.) and signals were detected by LAS-3000 mini CCD camera (Fuji Film). Primary antibodies used for immunoblotting were purchased from *e*Bioscience.

### Cytokine Analysis

Colonic IL-6, IL-1β, MCP-1, IL-17A were measured by ELISA according to the manufacturers’ instructions. ELISA kits were purchased from *e*Bioscience.

### Flow Cytometry

Single cell suspension was prepared from spleen by passing them through nylon mesh or colonic tissues as described above and stained with appropriate antibodies in PBS with 2% heat-inactivated fetal calf serum and 0.1% sodium azide on ice for 25 minutes. The antibodies used are following: fluorescein isothiocyanate (FITC)-labeled anti-murine Gr-1 (RB6-8C5, Biolegend), F4/80 (CI:A3-1, Biolegend) antibody, phycoerythrin (PE)-labeled anti-murine TNF-R1 (55R-286, Biolegend), TNF-R2 (TR75-89, Biolegend) antibody. These cells were fixed using PBS with 1% paraformaldehyde. For detecting apoptosis, double staining with annexin V and propidium iodide (PI) (eBioscience) was performed according to manufacturer’s instructions. Data collection and analysis were performed on a FACS Calibur flow cytometer using CellQuest software (Becton Dickinson).

### Real-time Reverse Transcriptase-polymerase Chain Reaction (RT-PCR)

RNA extracted from colon tissues was reverse-transcribed into cDNA using the SuperScript III First Strand cDNA synthesis system (Invitrogen). cDNA was synthesized from 0.5 µg RNA using random hexamer primers and SuperScript III(Invitrogen). Real-time RT-PCR was performed on a Bio-Rad iCycler to quantify mRNA levels. The primers for real-time were as follows: CXCL-1 forward primer 5′-ACT GCA CCC AAA CCG AAG TC-3′ and reverse primer 5′-TGG GGA CAC CTT TTA GCA TCT T-3′; CXCL-2 forward primer 5′-GCG CCC AGA CAG AAG TCA TAG-3′ and reverse primer 5′-AGC CTT GCC TTT GTT CAG TAT C-3′; CCL-3 forward primer 5′-TGT ACC ATG ACA CTC TGC AAC-3′ and reverse primer 5′-CAA CGA TGA ATT GGC GTG GAA-3′; GAPDH forward primer 5′-TGA AGG TCG GAG TCA ACG GAT TTG GT-3′ and reverse primer 5′-CAT GTG GGC CAT GAG GTC CAC CAC-3′. All reactions were performed in triplicate. The data were analyzed using Q-Gene software and expressed as fold change mean normalized expression (MNE) from control value. MNE is directly proportional to the amount of RNA of the target gene relative to the amount of RNA of the reference gene, GAPDH.

### Data Analysis

Student *t* test and 1-way analysis of variance (ANOVA) was used to determine significance, with *P*<0.05 considered significant. Kaplan-Meier was used for survival analysis with log rank *P*<0.05 to determine significance. Statistics were performed using SPSS 10.0 for Macintosh, and graphs were made on Deltagraph (SPSS, Chicago, I11.).

## Results

### TNF Signaling via TNF-R1 or 2 has Distinct Effects on Weight Loss and Mortality after DSS

**Figure 1:**
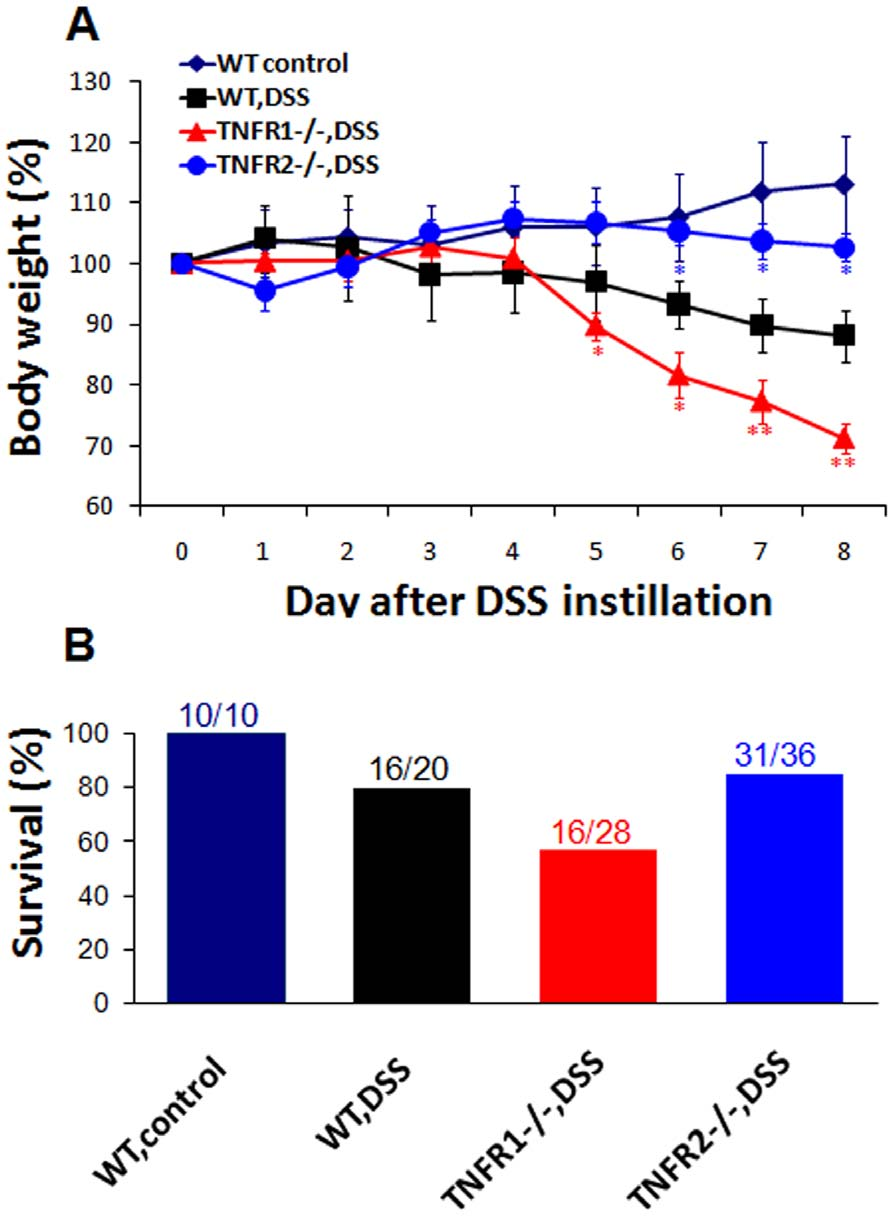
TNF-R1 or 2−/− mice have more or less weight loss and mortality after DSS. DSS colitis was established as described in Materials and methods. (A) Weight in each group was monitored daily at 8-day intervals. Weight was presented as percentage of the initial weight at day 0. (B) Survival rate was calculated at the end of experiments. Data are pooled from three independent experiments (n = 10–30 mice per group). *P<0.05, **P<0.01 vs WT mice after DSS.

Acute colitis was established in wild-type (WT) and TNF-R-knockout mice by continuous drinking of 3% DSS for eight days, respectively. WT mice lost an average of 11.8±4.3% of their baseline weight 8 days after DSS. TNF-R1−/− and TNF-R2−/− mice lost 28.8±2.6% and 4.4±2.3% at the same timepoint, respectively (Fig. 1A). Furthermore, in TNF-R1−/− group, acceleration of weight loss was initiated on day 5. In accord with this, at day 8, 57% (16/28) of TNF-R1−/−, 86% (31/36) of TNF-R2−/− mice had survived, whereas survival was 80% (16/20) in WT mice (Fig. 1B). Taken together, these results indicate that TNF-R1−/− mice exhibit more weight loss and mortality, while TNF-R2 deficiency attenuates the symptoms of colitis.

### Deficiency of TNF-R1 or 2 Affects Colon Injury Differently after DSS

**Figure 2:**
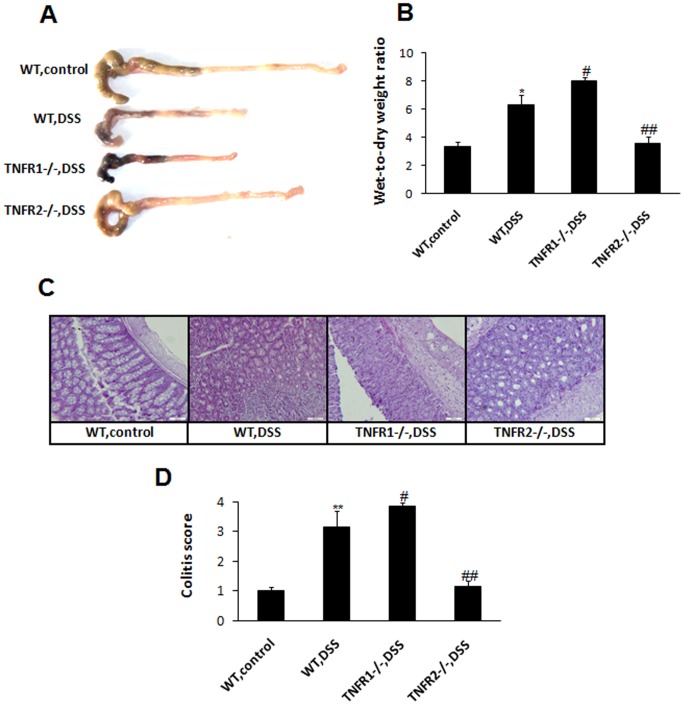
TNF-R1 or 2 deficiency exacerbates or ameliorates mucosal damage after DSS. Colons from each group were collected at day 8 after DSS instillation. (A) Macroscopic images of the colons are shown. (B) Colon edema was determined by wet-to-dry weight ratios. (C) Microscopic images of the colons are shown by haematoxylin and eosin staining. Magnification: ×200. (D) Colitis score was determined as described in Materials and methods. Data represent the mean±SD of eight to ten mice per group. *P<0.05, **P<0.01 vs WT control mice; #P<0.05, ##P<0.01 vs WT mice after DSS.

Next, the influence of TNF-R1 or 2 knockout on macroscopic and microscopic damage of colons after DSS was examined. WT mice displayed remarkable shortening and thickening of the colon 8 day after DSS (). TNF-R1 ablation led to more severe pathology of the target tissues. In contrast, the colon from TNF-R2−/− mice did not show considerable injury, resembling the colon of control mice (Fig. 2A). Moreover, wet-to-dry weight ratios of the colon from TNF-R1−/− group were significantly increased compared to WT mice after DSS (Fig. 2B). Whereas, the edema in TNFR2−/− colons was reduced obviously, which was comparable to the controls. Consistent with macroscopic changes, WT mice after DSS showed marked infiltration of inflammatory cells and ulceratic injury. TNF-R1 ablation rendered more severity of histopathological features of colitis (Fig. 2C). TNF-R2−/− colons, however, showed much less severity of intestinal damage. When quantified by histological system for evidence of inflammation and injury, these histological alterations were highly significant (Fig. 2D).

### Mice Lacking TNF-R1 or 2 have Higher or Lower Levels of MPO in Colons after DSS

**Figure 3:**
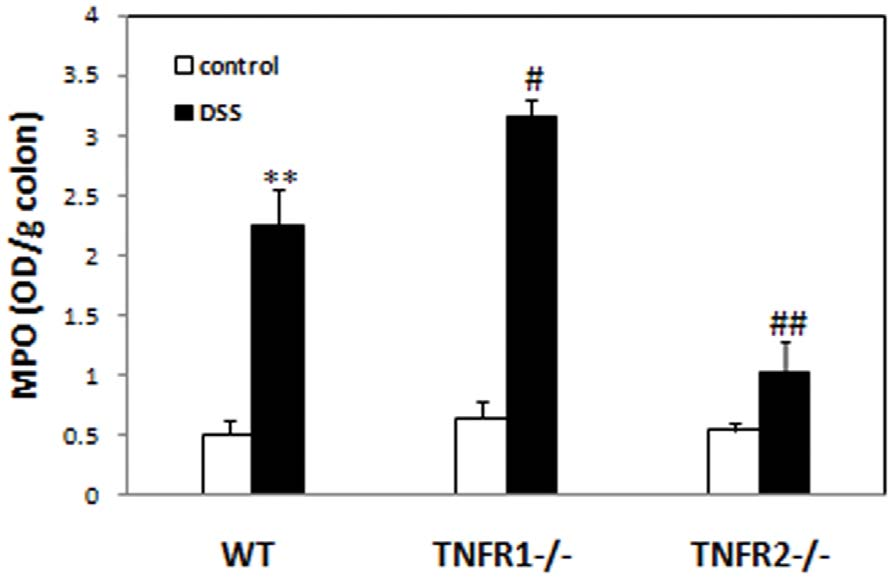
TNF-R1 or 2−/− mice have increased or reduced infiltrates of neutrophils into the colons after DSS. Levels of myeloperoxidase (MPO) in the colons of mice in each group were measured at day 8 after colitis induction. Data represent the mean±SD of four to six mice per group. **P<0.01 vs WT control mice; #P<0.05, ##P<0.01 vs WT mice after DSS.

Neutrophil infiltrate is a key event for initiation and promotion of inflammatory responses in many inflammatory diseases including IBD [17]–[19]. To determine mild or severe signs of colitis in mice lacking TNF-R1 or 2 are partially attributed to decreased or increased infiltrate of neutrophil, we detected the levels of MPO, indices for neutrophil infiltration, in the colon 8 days after DSS. Levels of MPO in the colons of WT colitis mice were significantly higher than control mice (Fig. 3). In mice lacking TNF-R1, the levels of colonic MPO were increased remarkably compared to WT mice after DSS. The levels of MPO in the colon of TNF-R2−/− mice, in contrast, were reduced to similar levels to those of control animals (Fig. 3).

### TNF-R1 or 2 Ablation Up-regulates or Down-regulates the Colonic Production of Proinflammatory Cytokines after DSS

**Figure 4:**
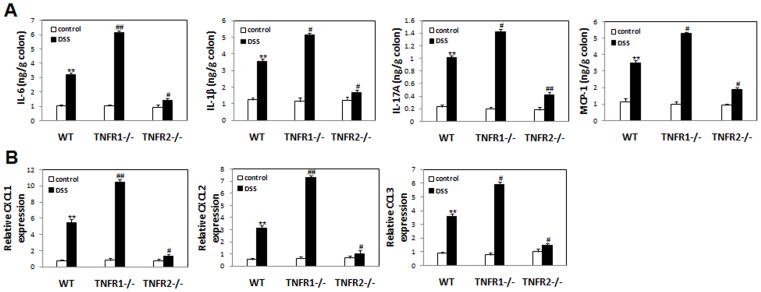
Lack of TNF-R1 or 2 increases or reduces proinflammatory mediator production in the colons after DSS. Colons were isolated and homogenized at day 8 after colitis induction. (A) IL-6, IL-1β, MCP-1, and IL-17A concentrations in the supernatants of homogenates were determined by ELISA. (B) mRNA were isolated from homogenates and CXCL1/2, CCL3 expression was examined by quantitative RT-PCR. Data represent the mean±SD of six to eight mice per group from two independent experiments. **P<0.05 vs WT control mice; #P<0.05, ##P<0.01 vs WT mice after DSS.

To determine whether opposite effects of TNF-R1 or 2 deficiency on pathological changes after DSS is associated with the alteration of expression of colitis-related proinflammatory cytokines and chemokines, the section of colons was dissected, homogenized and assayed at day 8 after DSS. Compared to WT colitis mice, lack of TNF-R1 resulted in significant up-regulation of IL-6, IL-1β, MCP-1, IL-17A levels in the colon tissues (Fig. 4A). In sharp contrast, the production of these cytokines in the lesions was decreased drastically (Fig. 4A). These results were in agreement with the data of colitis scores. Furthermore, the colonic expression of neutrophil-chemoattractant chemokines CXCL-1, CXCL-2, CCL-3 was increased in TNF-R1−/− mice, while was reduced in TNF-R2−/− counterparts (Fig. 4B). This result may explain the observation of the widespread infiltration of neutrophil in target tissues of TNF-R1-deficient mice instead of TNF-R2−/− mice.

### Lack of TNF-R1 Augments Systemic Inflammatory Responses

**Figure 5:**
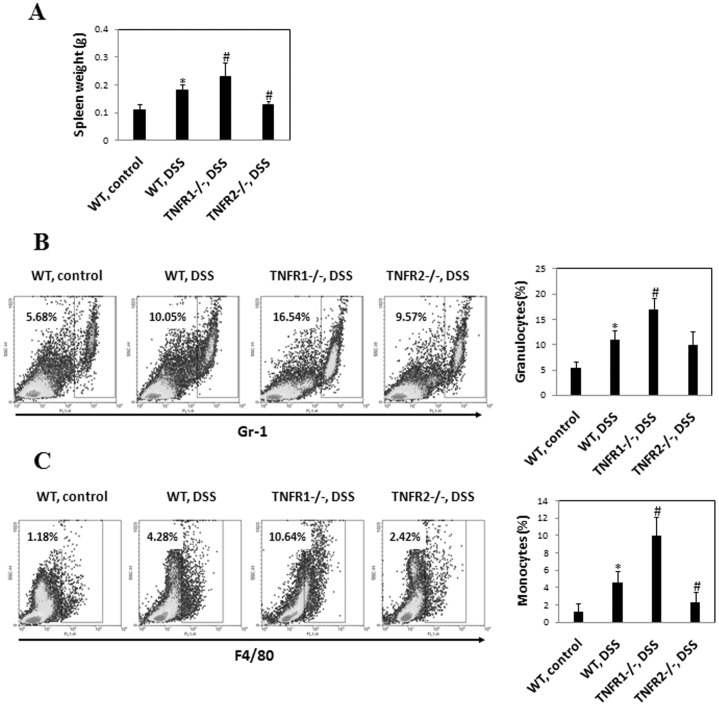
Systemic inflammatory response in TNF-R−/− and WT mice after DSS. (A) Spleen was isolated and weighed at day 8 after colitis induction. The percentage of granulocytes (Gr-1^+^) (B) or monocytes (F4/80^+^) (C) in the spleens were examined by flow cytometry. Representative plots were shown. Data represent the mean±SD of four to six mice per group from three independent experiments. *P<0.05 vs WT control mice; #P<0.05, ##P<0.01 vs WT mice after DSS.

It has been recognized that systemic inflammatory response is hallmark of DSS-induced colitis [20], [21]. Thus, we examined the influence of TNF-R1 or 2 knockout on the disease parameters. 8 days after DSS, WT mice exhibited visible splenomeagly as indicated by the weight of spleen. This effect was more pronounced in mice lacking TNF-R1 (Fig. 5A), indicating that TNF-R1 ablation led to augmented systemic inflammatory reaction. However, the weight of spleen in mice lacking TNF-R2 was comparable to controls, suggesting that absence of TNF-R2 inhibits local and systemic inflammatory responses after DSS.

Next, we defined the characteristics of this systemic inflammatory response induced by DSS administration. FACS analysis for frequency of granulocytes and monocytes in spleen was performed. The percentage of granulocytes (Gr-1^+^) was increased in WT, TNF-R1 or 2-knockout DSS-instilled mice compared to untreated controls (Fig. 5B), which was most pronounced in TNF-R1−/− group. Monocyte percentage, however, was sharply increased in WT and TNF-R1−/− groups, which was more significant in TNF-R1−/− mice. TNF-R2 deficiency did restrain the proliferation of monocytes in the spleen after DSS (Fig. 5C). These findings indicate that TNF signaling via TNF-R2 instead of TNF-R1 is required for systemic inflammatory responses.

### TNF-R1 or 2 Deficiency Reduces Anti-apoptotic Bcl-2 Expression and Enhances NF-κB Activity in Different Cell Types in the Colons after DSS

**Figure 6:**
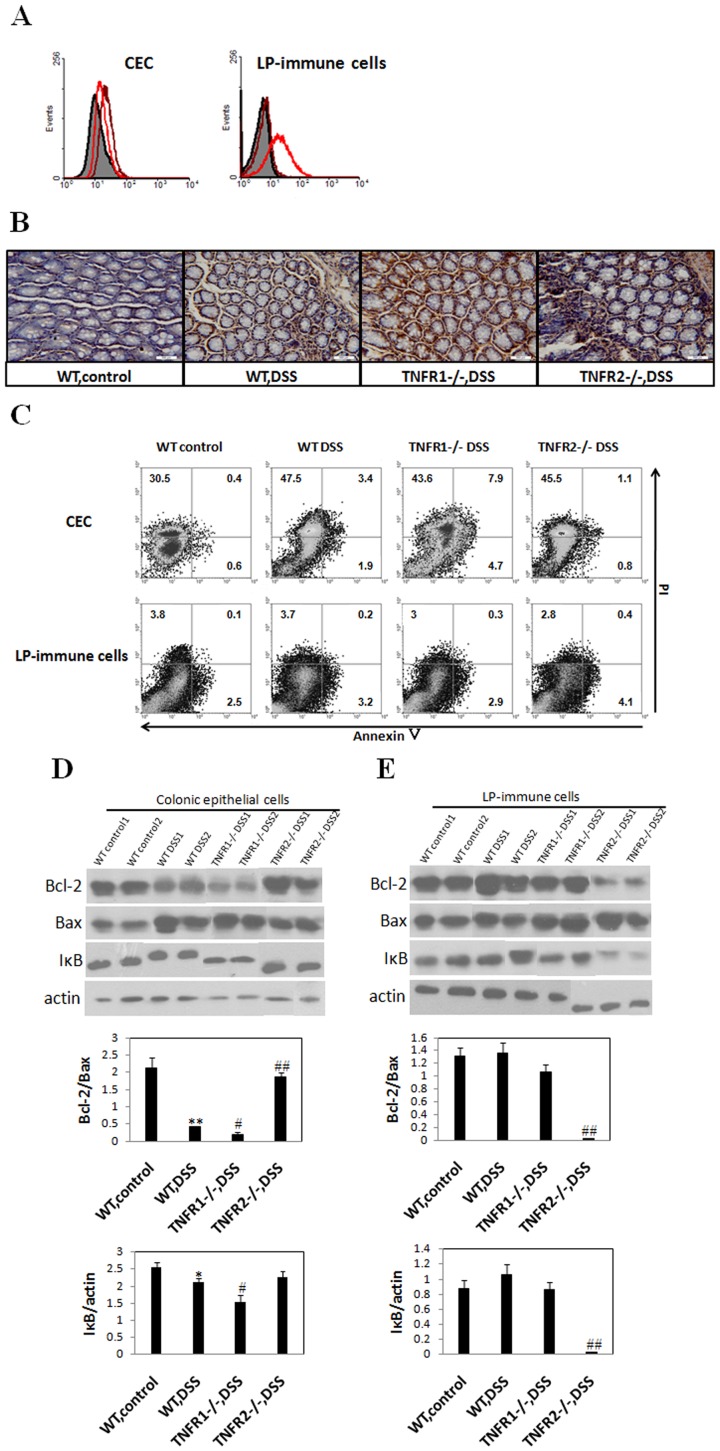
TNF-R1 or 2 ablation affects apoptosis of colonic epithelial cells or infiltrating immune cells differently after DSS. Colons were dissected at day 8 after colitis induction. (A) Colonic epithelial cells and LP-immune cells were isolated as described in Materials and methods. TNF-R1 (brown line) and 2 (red line) expression in these cells was examined by flow cytometry. (B) Apoptosis was determined by terminal deoxynucleotidyl transferase-mediated deoxyuridine triphosphate nick end-labeling (TUNEL) staining (brown nuclei). Magnification: ×400. Histology is representative for more than 10 mice per group. (C) Colonic epithelial cells and LP-immune cells were stained with annexin V and PI. Their apoptosis was detected by flow cytometry. (D,E) Colonic epithelial cells (D) and lamina propria-immune cells (E) were isolated respectively and the proteins were extracted. Bcl-2, Bax, and IκBα expression was examined by western blotting. Glyceraldehyde 3-phosphate dehydrogenase (GAPDH) was used as internal control. Changes in quantity of expression of these factors were determined by densitometric assays. Data are representative of three independent experiments. *P<0.05 vs WT control mice; #P<0.05, ##P<0.01 vs WT mice after DSS.

To determine the mechanisms underlying aggravated or attenuated intestinal damage in absence of TNF-R1 or 2 when DSS colitis was established, we speculate that lacking TNF signaling via TNF-R1 or 2 leads to alteration of apoptosis of colonic epithelial cells and/or lamina propria-residing immune cells. To address this issue, we first examined the expression of TNF-R1 and 2 on epithelial cells and LP-immune cells respectively. The result showed that TNFR1 was expressed only on CEC but not on LP-immune cells (Fig. 6A). Subsequently, colon samples from WT and TNF-R knockout groups were stained using the TUNEL methods. The apoptotic cells in colonic mucosa of WT control mice were almost invisible. Upon the establishment of DSS-induced colitis, numerous apoptotic cells in the epithelium were observed (Fig. 6B). In agreement with the grade of mucosal damage, TNF-R1−/− mice, which had higher colitis scores, showed significant increase of apoptotic epithelial cells (Fig. 6B). Interestingly, when TUNEL staining was done in the colon sample of DSS-instilled TNF-R2−/− mice, the enhanced apoptosis of LP-mononuclear immune cells rather than epithelial cells was seen (Fig. 6B). To further address this issue, we detected the apoptosis of colonic epithelial cells and LP-immune cells by annexin V/PI staining respectively. Significantly increased apoptosis of colonic epithelial cells was observed in TNF-R1−/− mice after colitis induction. While, TNFR2 deficiency led to increased number of apopototic LP-immune cells, instead of CEC, upon colitis establishment (Fig. 6C).

Next, we examine whether increase in apoptosis of colonic epithelial cells or LP-immune cells in TNF-R1or 2 knockout mice after DSS is associated with reduced anti-apoptotic signals. The results showed that DSS instillation drastically reduced the expression of anti-apoptotic factor Bcl-2 in CEC of WT mice (Fig. 6D). This effect was more significant in TNF-R1−/− mice (Fig. 6D). Consistent with the data from TUNEL staining, reduction of Bcl-2 contents was found in LP-immune cells of TNFR2−/− mice (Fig. 6E). Furthermore, densitometry analysis showed that the ratio of Bcl-2/Bax in TNF-R1−/− or TNF-R2−/− mice was pronouncedly lower than in WT mice after DSS (Fig. 6E). Further, to elucidate the effects of TNF-R deficiency on NF-κB activity, we detected IκBα expression in the CEC or LP-immune cells after DSS. The amounts of IκBα in CEC for TNF-R1−/− mice or in LP-immune cells for TNF-R2−/− mice were much lower than those in WT DSS-treated mice (Fig. 6D and E), indicating that NF-κB activity was enhanced in CEC and LP-immune cells respectively. Thus, cell type-specific reduction in anti-apoptotic proteins and enhanced NF-κB activity may account for exacerbation or amelioration of colitis in TNFR1 or 2−/− mice after DSS.

## Discussion

TNF plays a critical role in the immunopathogenesis of IBD. Blocking TNF using specific monoclonal antibody has been identified to be beneficial for treatment of patients suffering from IBD [22]. Blockade of TNF, however, has been associated with the reactivation of tuberculosis and the possible development of other opportunistic infections such as histoplasmosis, listeriosis and pneumocystis [23]. Thus, it is very necessary to dissect the role of TNF signaling via TNF-R1 or 2 in experimental colitis, given that the beneficial outcome of complete blocking TNF may be achieved from blocking the signaling from only one of the two receptors. In this report, we provide evidence that TNF signaling via TNF-R1 or 2 has opposite effects on the pathogenesis of DSS-induced colitis. That is, TNF-R1-mediated signaling ameliorated the severity of colitis, while TNF-R2-mediated signaling was detrimental for the course of colitis. The data presented in this paper strongly support that blocking the interaction of TNF with its receptor 2 alone is sufficient to treat IBD.

It is worthy to note that the data presented in our study are incompletely consistent with a previous report that TNF signaling via TNF-R1 or 2 is redundant for the pathogenesis of DSS colitis in C57Bl/6 mice [13]. This discrepancy may be resolved by the fact that different mouse strains were used in these studies, as BALB/c strains used in our study are well known to be predisposed to a Th2 response, while C57Bl/6 strains do not. Interestingly, TNF signaling via TNF-R1 or 2 also appears to have distinct effects on different models of experimental colitis. Our previous study demonstrated that deficiency of TNF-R1 or 2 attenuated intestinal damage and improved the symptoms of TNBS-instilled colitis [12]. This may be explained by that different mechanisms are responsible for the initiation and progression of DSS and TNBS-induced experimental colitis respectively [24]. Interestingly, it is well accepted that intestinal inflammation induced by TNBS in BALB/c strains displays human CD-like features, while the pathogenesis of DSS colitis resembles ulcerative colitis in human [24]. Therefore, these data have implications for potential regimens targeting TNF-R1 or 2 to treat CD or UC in clinic.

The harmful or beneficial outcome of lack of TNF-R1 or 2 on DSS-induced colitis is tightly related to alteration of production of proinflammatory cytokines in the attacked tissues. This result indicates that interference with TNF signaling affects other putatively colitis-related cytokine expression, for example IL-6. Indeed, TNF can directly induce IL-6 production by NF-κB-, NF-IL-6-, and AP-1-dependent mechanisms [25], it also can facilitate the recruitment and survival of proinflammatory immune cells capable of IL-6 production [3]. The data in our paper extend these findings. IL-6 production induced by TNF is mediated mainly by TNF signaling via TNF-R2, as lacking TNF-R1 led to significant up-regulation of IL-6 expression in the colon, while TNF-R2 deficiency did not. Furthermore, the mutual regulation of TNF-R1 and 2-mediated signaling is orchestrated on proinflammatory cytokine production thereby signs of colitis, because TNF signaling via TNF-R1 dampens TNF-R2-mediated pathogenic responses, as shown by the fact that ablation of TNF-R1 dramatically aggravated TNF-R2-mediated colonic inflammation. The details, however, need further investigation.

One of the hallmarks of intestinal inflammation is the infiltration of neutrophils into colons and release of large amounts of oxgen radicals [18]. Our results showed significant increase of neutrophil infiltrates in sites of inflammation in TNF-R1−/− mice when DSS-induced colitis was established. Whereas, TNF-R2-deficient mice exhibited reduced infiltration of neutrophils, which may account for blunted intestinal inflammatory responses. Thus, we believe that the absence of TNF signaling via TNF-R1 or 2 affects neutrophil recruitment differently into colonic tissues upon the establishment of colitis. Intriguingly, these results are incompletely consistent with our previous study showing that TNF-R1 or 2−/− TNBS-instilled mice displayed restricted recruitment of neutrophils into inflamed colons, which may reflect the difference in the virtue of inflammation induced by DSS and TNBS. Increased infiltration of neutrophil in the colon of TNF-R1−/− colitic mice may be due to up-regulated expression of several putatively neutrophil-chemoattractant chemokines in local compartment, although the underlying mechanisms remain unclear [26]. Notably, it has been reported that TNF is able to induce integrin CD11b/CD18 up-regulation on human neutrophils, enhance cell adhesion and recruit as well as retain leukocytes in inflammatory sites [27]. Thus, TNF signaling may affect directly and indirectly the recruitment of neutrophils into inflamed tissues, thereby provoke or resolve inflammation in TNF-R1 or 2-deficient mice.

Mucosal damage is a key event for DSS-induced colonic inflammation. TNF is an important factor in this process, as in pathological settings TNF is released largely and has deleterious effects on epithelial cells [1]. Our results showed that DSS administration led to apoptosis of colonic epithelial cells by reducing expression of anti-apoptotic protein Bcl-2 and enhancing NF-κB activity. Lack of TNF signaling via TNF-R1 could accelerate this apoptosis-promoting process and rendered increased death of epithelial cells, finally resulted in more severity of mucosal injury. Interestingly, the absence of TNF signaling via TNF-R2 influenced mildly apoptosis of CEC, but drastically enhanced programmed cell death of LP-immune cells, which may account for resolution of inflammation in TNF-R2−/− mice. These data indicate that, in the setting of intestinal inflammation, colonic epithelial cells and inflammatory immune cells have different susceptibility to TNF-mediated apoptosis through TNF-R1 or 2. This concept is supported by the previous studies showing that TNF-R1 is dominantly functional to mediate TNF signaling in endothelial cells [28], myofibroblast [29], and infected host cells [30], while TNF function on immune cells is predominantly through TNF-R2 [31], [32]. Although the pathways involved in TNF-mediated apoptosis of CEC and LP-immune cells need to be elucidated further, the present data indicate that enhanced apoptosis of CEC or LP-immune cells is one of the reasons for exacerbated or ameliorated inflammation in TNF-R1 or 2−/− mice after DSS.

In the current study, we demonstrated an opposite role of TNF-R1 or 2 in the course of DSS-induced colitis, which is associated with alteration in several parameters, including infiltration of granulocytes, release of proinflammatory mediators and apoptosis of epithelial cells as well as inflammatory immune cells. These data suggest that targeting TNF-R2 rather than TNF-R1 specifically might be an alternative approach to therapy for patients with UC, considering that TNF is a key factor in host defense against microbe infection [33]. Although TNF-R2 deficiency failed to expand monocyte population specifically after DSS, TNF signaling via TNF-R2 had minor role in anti-mycobacterial immunity [34]. So blockade of TNF-R2 specifically may be an alternative and promising approach to treat IBD. The studies in TNBS or DSS-induced experimental colitis suggest that the exact role of TNF signaling via TNF-R1 or 2 may depend on model of colitis and mouse strains used and need to be interpreted carefully. Further investigation in other models and in humans is warranted.
